# Comparison of visual quality after SMILE correction of low-to-moderate myopia in different optical zones

**DOI:** 10.1007/s10792-023-02771-6

**Published:** 2023-07-15

**Authors:** Cong Zhou, Ying Li, Yinghan Wang, Qiuyang Fan, Lili Dai

**Affiliations:** Department of Ophthalmology, The Second Affiliated Hospital of Harbin Medical University, No. 246, Xuefu Road, Nangang District, Harbin, 150086 China

**Keywords:** Small-incision lenticule extraction, Low-to-moderate myopia, Optical zone, High-order aberration, Visual quality

## Abstract

**Objective:**

To compare the effects of different optical zones for small-incision lenticule extraction (SMILE) on postoperative visual quality in low-to-moderate myopia.

**Methods:**

This retrospective case–control study involved patients who underwent SMILE using two optical-zone diameters: 6.5 mm (50 patients, 100 eyes) and 6.8 mm (50 patients, 100 eyes). Uncorrected visual acuity (UCVA), best corrected visual acuity, spherical equivalent (SE), corneal higher-order aberrations (HOAs), and subjective visual-quality questionnaire scores were assessed.

**Results:**

Postoperatively, UCVA and SE did not differ between the two groups (*P* > 0.05). In both groups, corneal HOAs, spherical aberration, and coma significantly increased at 1 and 3 months postoperatively (*P* < 0.05), while trefoil was unchanged after surgery (*P* > 0.05). Corneal HOAs, spherical aberration, and coma significantly differed between the groups at 1 and 3 months (*P* < 0.05), while trefoil did not (*P* > 0.05). Visual-quality scores were higher in the 6.8 mm group than in the 6.5 mm group at 1 month (*P* = 0.058), but not at 3 months (*P* > 0.05). In both groups, subjective scores significantly decreased at 1 month (*P* < 0.05) and gradually returned to the preoperative level at 3 months (*P* > 0.05). The subjective visual-quality scores were negatively and positively correlated with pupillary and optical-zone diameter, respectively (*P* < 0.05 for both). Objective visual-quality indicators (HOAs, spherical aberration, and coma) were negatively correlated with optical-zone diameter (*P* < 0.05) but not pupillary diameter (*P* > 0.05).

**Conclusion:**

SMILE in different optical zones effectively corrected low-to-moderate myopia. The larger the optical-zone diameter, the better the early postoperative visual quality.

## Introduction

Small-incision lenticule extraction (SMILE) is a novel approach in corneal refractive surgery that offers the advantages of being minimally invasive, flapless, and accurate. SMILE has shown significant effectiveness, safety, stability, and predictability in correcting different degrees of myopia and myopic astigmatism [1–4]. However, some patients report having unclear vision in the early daytime and uncomfortable symptoms at night such as glare, halos, and decreased night vision during the early postoperative period. These symptoms tend to be common among patients with small optical-zone diameters, especially optical-zone diameters smaller than the pupillary diameter in a dark environment [5]. To correct this, some authors used larger optical-zone diameters, but the postoperative visual quality remained unsatisfactory in some patients [6].

The optical zone in SMILE surgery refers to the diameter of the corneal incision. When the pupillary diameter is larger than the planned optical zone (i.e., the cornea cut), the light passing through the peripheral zone will produce aberrations, thereby reducing the image quality on the retina and the postoperative satisfaction of the patient. Corneal higher-order aberrations (HOAs) are an important consideration in evaluating visual quality after corneal refractive surgery, as these aberrations will lead to symptoms of decreased visual quality, such as ghost images and glare [7, 8]. To address this, some authors have applied laser-assisted in situ keratomileusis (LASIK) [9], laser subepithelial keratomileusis (LASEK) [10], and femtosecond laser-assisted in situ keratomileusis (FS-LASIK) [11, 12] in corneal refractive surgery, and found that an appropriate increase in the corneal optical-zone diameter effectively reduced postoperative corneal aberrations and improved postoperative outcomes. However, to date, few reports have investigated the early visual quality after SMILE using different optical zones, and their results were varied [13, 14]. In the current study, we aimed to evaluate the subjective and objective visual quality in the early postoperative period after SMILE using two different optical-zone diameters in order to provide a reference for personalized surgery.

## Materials and methods

### Subjects and groups

This retrospective case–control study involved a total of 100 patients (200 eyes) who underwent SMILE surgery at the Second Affiliated Hospital of Harbin Medical University between October 2018 and July 2019. The inclusion criteria were as follows: age, 18 years or older; no history of eye disease or trauma; transparent cornea without haze or macula; soft contact lens wearers who had not worn contacts for more than 2 weeks or rigid contact lens wearers who had not worn contacts for more than 4 weeks; and annual increase in refractive error < 0.50 D within 2 years. The exclusion criteria were as follows: corneal thickness did not meet the requirements for surgery, keratoconus or suspected keratoconus, and other serious ocular or systemic diseases. The patients were divided into 2 groups based on the preoperatively planned optical-zone diameter: a 6.5 mm group (50 patients, 100 eyes) and a 6.8 mm group (50 patients, 100 eyes). The selection of the optical-zone diameter depended on the patients’ dark-room pupillary diameter and corneal thickness. All operations were performed independently by YL. This study was approved by the ethics committee of The Second Affiliated Hospital of Harbin Medical University Review Board (KY2021–011), and this trial was registered in the Chinese Clinical Trial Registry (ChiCTR2100044212). All patients signed informed consent forms before the surgery.

### Routine examination

The preoperative examination included the following: slit-lamp microscope examination; computerized, comprehensive, and cycloplegic optometry; measurement of the uncorrected visual acuity (UCVA) and best corrected visual acuity (BCVA); central corneal thickness (CCT); corneal topography; intraocular pressure; axis length; fundus examination with 3 mirrors after mydriasis; and pupillary diameter in bright and dark environments. Aberrations of the anterior corneal surface under the condition of a pupillary diameter of 6 mm were detected using a Sirius 3D corneal topographic anterior-segment analysis system, which can detect HOAs, spherical aberration, coma, and trefoil. The results were presented as root mean squares (RMSs). We also used a self-reported subjective visual-quality questionnaire that was adjusted according to the Ocular Surface Disease Index (OSDI) score and included the patients’ complaints. The questionnaire survey addressed the following 17 subjective visual-quality characteristics: glare, halo, light sensitivity, blurred vision, diplopia, ghost images, vision distortion, field of vision, any effect on vision while driving at night, headache, eye pain, red eyes, dry eye, photophobia, burning sensation when shedding tears, and foreign body sensation. Each item was scored on a scale from 1 to 4 points, which indicated none, mild, moderate, and severe symptoms, respectively. The lower the score, the more severe or uncomfortable the symptom. The average value of all the item scores was taken as the total subjective score.

### Postoperative review

All patients were followed up at 1 day, 1 month and 3 months after the surgery. Slit-lamp microscopy was performed, and UCVA, computerized refraction, intraocular pressure, Sirius corneal topography (Costruzione Strumenti Oftalmici, Florence, Italy), and corneal aberrations were assessed at each follow-up visit. The self-reported subjective visual-quality questionnaire was also completed at each visit.

### Experimental data collection

UCVA and BCVA were examined using standard logarithmic visual acuity charts, and the results were converted to logMAR during data analysis. Spherical and cylindrical refraction and the self-reported subjective visual-quality evaluation questionnaire scores were also examined. The RMS values of HOAs, spherical aberration, coma, and trefoil on the anterior corneal surface under the condition of a 6 mm pupillary diameter were measured using the Sirius 3D corneal topographic mapping and anterior-segment analysis system.

### Surgical technique

All surgeries were performed by the same experienced surgeon from the Second Affiliated Hospital of Harbin Medical University. The VisuMax 3.0 femtosecond laser system (Zeiss, Germany) was used in the standard mode to perform the SMILE procedure. The laser settings were as follows: frequency, 500 kHz; pulse energy, 130 nJ; CCT, 110–120 μm; and target lenticule (optical zone) diameter, 6.5 or 6.8 mm, depending on the preoperative corneal thickness and the refractive error to be corrected.

### Statistical analysis

SPSS Version 23.0 (IBM company, Chicago, Illinois, USA) was used for statistical analysis. First, the Shapiro–Wilk test was used to test the normality of the distribution of measurement data. Data with a normal distribution were expressed as mean ± standard deviation, and data with a skewed distribution were presented as median (P_25_, P_75_). Because visual quality is affected by both eyes, we used a typical two-level structure for analysis with a mixed model. According to the Akaike Information Criterion, a smaller value indicated a better model; it also showed that the model with repeated effects (mixed-effect model) fitted the data considerably well and had the best co-variance structure. The mixed-effect model was used to compare differences in measurement data between the two groups and to compare the pre- and postoperative data. The correlation of postoperative visual quality with pupillary diameter and optical-zone diameter was analyzed using partial correlation. *P* < 0.05 was considered statistically significant.

## Results

### Preoperative characteristics

The patients included 57 men (114 eyes) and 43 women (86 eyes) with an average age of 23.00 ± 4.84 years (range, 18–39 years). Their refractive errors were as follows: spherical, − 1.00 to − 5.25 D; astigmatism, 0 to − 2.00 D; spherical equivalent (SE), − 1.75 to − 6.00 D, and average refractive error, − 4.00 ± 0.86 D. The preoperative data, such as age, refractive error, CCT, pupillary diameter, intraocular pressure, and BCVA, did not significantly differ between the 2 groups (*P* > 0.05; Table 1).

**Table 1:** Preoperative characteristics of participants in the 6.5 mm and 6.8 mm groups

Variable	6.5 mm (n = 100 eyes)	6.8 mm (n = 100 eyes)	*P* value
Age (years)	21.00 (19.00, 26.00)	18.00 (22.00, 28.00)	0.736
Gender (female)	48 (48%)	38 (38%)	0.153
Spherical (D)	− 3.78, 0.10 (− 3.98, − 3.58)	− 3.61, 0.10 (− 3.81, − 3.40)	0.225
Cylinder (D)	− 0.64, 0.06 (− 0.77, − 0.52)	− 0.58, 0.06 (− 0.70, − 0.46)	0.450
SE (D)	− 4.10, 0.10 (− 4.31, − 3.90)	− 3.89, 0.10 (− 4.10, − 3.69)	0.158
HOAs (μm)	0.42, 0.02 (0.39, 0.45)	0.42, 0.02 (0.39, 0.46)	0.839
CCT (μm)	541.06, 3.48 (533.16, 546.96)	546.91, 3.48 (540.01, 553.81)	0.167
Pupillary diameter (mm)	6.46, 0.09 (6.27, 6.64)	6.60, 0.09 (6.41, 6.78)	0.283
IOP (mm Hg)	18.17,0.31 (17.55, 18.79)	17.92, 0.31 (17.30, 18.54)	0.572
BCVA (logMAR)	− 0.00, 0.00 (− 0.01, 0.00)	0.00, 0.00 (− 0.00, 0.01)	0.338

Age is presented as median (P25, P75). The other variables are presented as least squares means, standard errors, and 95% confidence intervals

*SE* spherical equivalent, *D* diopters, *CCT* central corneal thickness, *IOP* intraocular pressure, *BCVA* best corrected visual acuity, *logMAR* logarithm of the minimal angle of resolution

### Postoperative visual acuity and refractive error

**Fig. 1:**
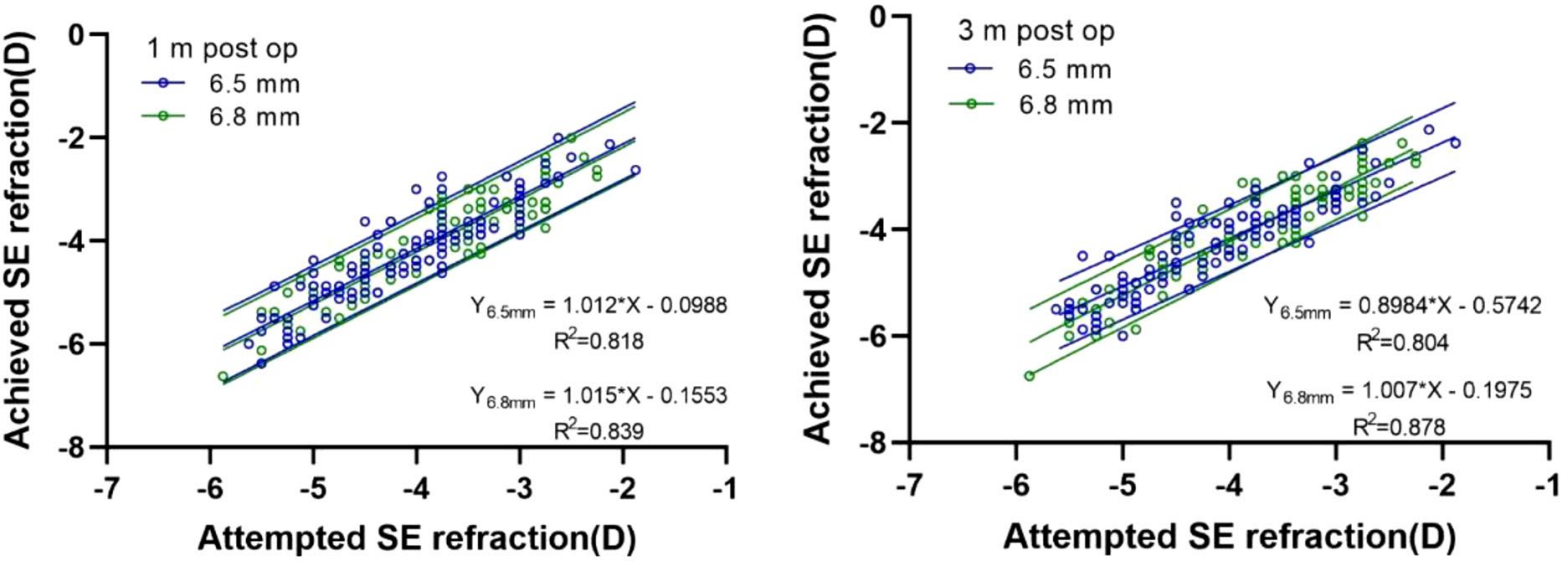
Correlation between the attempted SE refraction and the SE refraction achieved at 1 month and 3 months after SMILE surgery in the 6.5 mm and 6.8 mm groups. SE, spherical equivalent; SMILE, small-incision lenticule extraction; D, diopters

The UCVA and SE did not significantly differ between the two groups at 1 month or 3 months after the surgery (*P* > 0.05; Table 2), In both groups, the UCVA (logMAR) was ≥ 1.0 at 1 month and 3 months after the surgery, which was greater than or equal to the preoperative BCVA. Furthermore, the actual corrected SE (diopters) at 1 month and 3 months after the surgery was within the preoperative corrected SE range (± 1.00 D) in both groups, indicating a stable state (Fig. 1).

**Table 2:** Comparison of UCVA and SE at 1 and 3 months after surgery between the 6.5 mm and 6.8 mm groups

Group	UCVA (logMAR)		SE (D)	
	1 month	3 months	1 month	3 months
6.5 mm	0.09, 0.01 (0.08, 0.10)	0.10, 0.01 (0.08, 0.11)	0.15, 0.05 (0.06, 0.24)	0.16, 0.05 (0.06, 0.25)
6.8 mm	0.09, 0.01 (0.08, 0.11)	0.11, 0.01 (0.10, 0.12)	0.21, 0.05 (0.12, 0.31)	0.23, 0.05 (0.14, 0.32)
*P* value	0.889	0.145	0.311	0.282

Variables are presented as least squares means, standard errors, and 95% confidence intervals

*UCVA* uncorrected visual acuity, *SE* spherical equivalent, *D* diopters, *logMAR* logarithm of the minimal angle of resolution

### Changes in RMS values of corneal aberrations before and after surgery

**Fig. 2:**
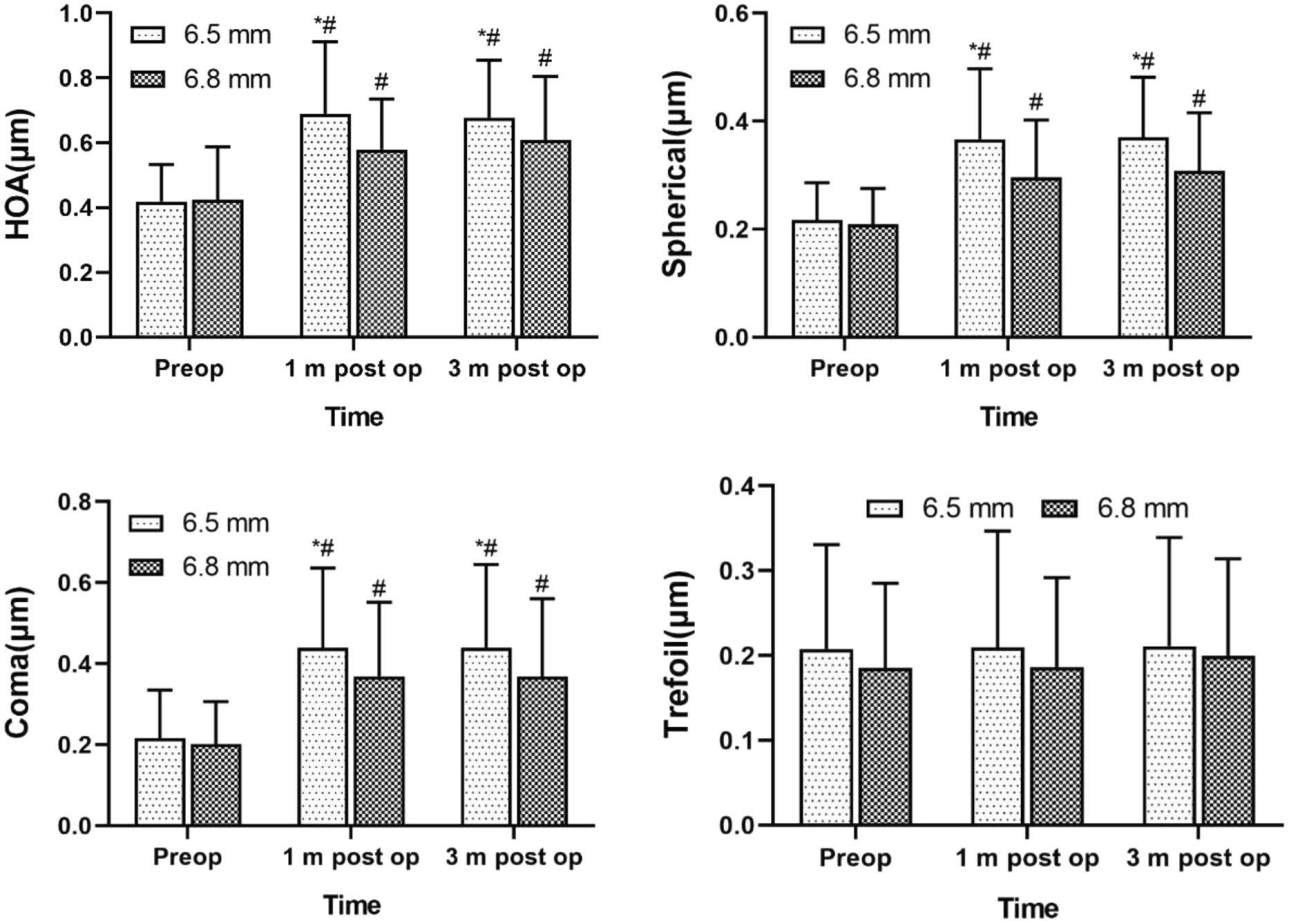
Comparison of aberrations in the 6.5 mm and 6.8 mm groups before, and 1 month and 3 months after the surgery.*Indicates a significant difference from the 6.8 mm group at the same point; #indicates a significant difference from the preoperative value

The preoperative RMS values of HOAs, spherical aberration, coma, and trefoil did not differ between the 2 groups (*P* > 0.05). The RMS values of corneal HOAs, spherical aberration, and coma significantly differed between the 2 groups at 1 month and 3 months after the surgery (*P* < 0.05), while the postoperative RMS values of trefoil did not differ between the 2 groups (*P* > 0.05). In both groups, the RMS values of corneal HOAs, spherical aberration, and coma at 1 and 3 months after the surgery significantly differed from the preoperative data (*P* < 0.05), while the postoperative RMS values of trefoil did not significantly differ from its preoperative values (*P* > 0.05; Table 3, Fig. 2).

**Table 3:** Comparison of aberrations in the 6.5 mm and 6.8 mm groups before and after surgery

Group	HOAs (μm)	Spherical (μm)	Coma (μm)	Trefoil (μm)
*Preoperative*				
6.5 mm	0.42, 0.02 (0.38, 0.46)	0.22, 0.01 (0.19, 0.24)	0.22, 0.02 (0.17, 0.26)	0.21, 0.01 (0.18, 0.24)
6.8 mm	0.41, 0.02 (0.37, 0.45)	0.21, 0.01 (0.19, 0.23)	0.20, 0.02 (0.16, 0.24)	0.19, 0.01 (0.16, 0.21)
*P* value	0.826	0.720	0.621	0.234
*1 month postoperative*				
6.5 mm	0.69, 0.02 (0.65, 0.73)^*^	0.37, 0.01 (0.34, 0.39)^*^	0.44, 0.02 (0.40, 0.48)^*^	0.20, 0.01 (0.18, 0.23)
6.8 mm	0.58, 0.02 (0.54, 0.62)^*^	0.30, 0.01 (0.27, 0.32)^*^	0.37, 0.02 (0.33, 0.41)^*^	0.18, 0.01 (0.16, 0.21)
*P* value	0.001	< 0.001	0.024	0.305
*3 months postoperative*				
6.5 mm	0.68, 0.02 (0.64, 0.72)^*^	0.37, 0.01 (0.35, 0.39)^*^	0.44, 0.02 (0.40, 0.48)^*^	0.21, 0.01 (0.19, 0.24)
6.8 mm	0.61, 0.02 (0.57, 0.65)^*^	0.31, 0.01 (0.28, 0.33)^*^	0.37, 0.02 (0.32, 0.41)^*^	0.20, 0.01 (0.17, 0.22)
*P* value	0.012	< 0.001	0.018	0.407

Variables are presented as the least squares means, standard errors, and 95% confidence intervals

*HOA* higher-order aberration

Compared with the preoperative values, ^*^P < 0.05

### Preoperative and postoperative subjective visual-quality scores

The subjective visual-quality scores were similar in both study groups before the surgery (*P* > 0.05), differed between the two groups at 1 month after the surgery (*P* = 0.058, close to 0.05), and did not differ between the two groups at 3 months after the surgery (*P* > 0.05). In both groups, the subjective visual-quality scores were significantly lower at 1 month after the surgery than before the surgery (*P* < 0.05), but gradually increased to the preoperative level at 3 months after the surgery (*P* > 0.05; Table 4).

**Table 4:** Comparison of subjective visual-quality scores between the 6.5 mm and 6.8 mm groups before surgery, and 1 month and 3 months after surgery

Time point	6.5 mm	6.8 mm	*P* value
Preoperative	3.86, 0.02 (3.82, 3.90)	3.75, 0.02 (3.70, 3.79)	0.997
1 month postoperative	3.85, 0.02 (3.81, 3.89)^*^	3.86, 0.02 (3.82, 3.90)^*^	0.058
3 months postoperative	3.80, 0.02 (3.76, 3.84)	3.86, 0.02 (3.82, 3.90)	0.814

Variables are presented as the least squares means, standard errors, and 95% confidence intervals

Compared with the values before surgery, ^*^P < 0.05

### Correlation of postoperative subjective and objective visual quality with pupillary diameter and optical-zone diameter

The overall subjective visual-quality scores at 1 and 3 months after SMILE showed a significant negative correlation with the pupillary diameter (correlation coefficients, − 0.349 and − 0.461, respectively). In contrast, HOAs, spherical aberration, coma, and trefoil were not correlated with the pupillary diameter at 1 and 3 months after SMILE. The overall subjective visual-quality score at 1 month after SMILE was significantly and positively correlated with the optical-zone diameter (correlation coefficient, 0.162). The RMS values of the objective indicators of visual quality, such as HOAs, spherical aberration, and coma, at 1 month and 3 months after the operation were significantly and negatively correlated with the optical-zone diameter, while trefoil was not significantly correlated with the optical-zone diameter at 1 and 3 months after the operation (Table 5).

**Table 5:** Correlation of visual-quality parameters with pupillary diameter and optical-zone diameter in 100 patients (200 eyes) with refractive errors at different time points after SMILE surgery

Time point	SVQ	HOAs (μm)	Spherical (μm)	Coma (μm)	Trefoil (μm)
*Pupillary diameter*					
1 month postoperative	− 0.349^**^	0.016	0.094	0.079	0.018
3 months postoperative	− 0.461^**^	0.089	0.140	0.082	0.125
*Optical-zone diameter*					
1 month postoperative	0.162^*^	− 0.432^**^	− 0.597^**^	− 0.247^**^	− 0.004
3 months postoperative	0.072	− 0.460^**^	− 0.543^**^	− 0.372^**^	− 0.008

The correlation coefficient was adjusted for gender, age and eye position

*SVQ* subjective visual quality, *HOAs* higher-order aberrations

^**^P < 0.01; ^*^P < 0.05

## Discussion

In the present study, we performed SMILE surgery using 2 different optical-zone diameters, and compared and analyzed the clinical effects. The results showed that the UCVA did not differ between the two groups at 1 month and 3 months after the surgery (*P* > 0.05), indicating that SMILE was effective in correcting low-to-moderate myopia and myopic astigmatism within a certain optical zone. At 1 month and 3 months after the surgery, the UCVA (logMAR ≥ 1.0) equaled or exceeded the preoperative BCVA, indicating that the surgery was highly effective in both groups. The SE of the patients in both groups was within the expected correction range (± 1.00 D) at 1 month and 3 months after the surgery and remained stable, indicating that SMILE surgery in different optical zones had high predictability.

After corneal refractive surgery, changes in corneal morphology and corneal thinning lead to corneal HOAs, which are a key factor affecting postoperative visual quality. However, the reported changes in corneal wavefront aberrations after SMILE are not consistent. Siedlecki et al. [15] followed up 197 patients (394 eyes) for an average of 2 years, and found that the total HOAs, coma, spherical aberration, and trefoil increased significantly after SMILE. However, Xia et al. [16] followed up patients for 7 years after SMILE, and found no significant changes in HOAs, spherical aberration, horizontal coma, and trefoil before and after SMILE surgery. In our study, under the condition of a pupillary diameter of 6 mm, the RMS values of corneal HOAs, spherical aberration, and coma were significantly higher postoperatively than preoperatively (*P* < 0.05), but there was no significant difference in trefoil before and after SMILE surgery, which is consistent with previously reported changes in corneal wavefront aberrations after SMILE [17, 18].

Our study showed that a larger optical-zone diameter during SMILE was associated with fewer postoperative HOAs. Specifically, the RMS values of HOAs, spherical aberration, and coma were significantly lower in the 6.8 mm group than in the 6.5 mm group (*P* < 0.05), which is consistent with previous results [13]. Studies have compared the effects of different optical zones in other types of corneal refractive surgery on postoperative HOAs. Ozulken and Gokce [11, 19] compared the effects of different optical-zone diameters for FS-LASIK and photorefractive keratectomy on HOAs, and found that compared with a 6.5 mm optical zone, optical zones > 7 mm were associated with significantly less spherical aberration (*P* < 0.05); the authors further showed that the optical-zone diameter mainly affected the increase in postoperative spherical aberration. Zhao et al. [12] compared HOAs of the cornea under different pupillary diameters (3, 5, and 7 mm) after FS-LASIK with 6.0 mm and 6.5 mm optical zones, and the results showed that for pupillary diameters < 7 mm, the total HOAs and spherical aberrations were significantly higher in the 6.0 mm group than in the 6.5 mm group, whereas no significant differences were found in coma. The difference between the above study and our study may be related to differences in the aberration-inspection equipment and operating techniques. However, another study has suggested that SMILE not only corrected refractive error but also reduced spherical aberration, coma, and trefoil to a certain extent, and that its outcomes did not significantly differ between the small-aperture (6.1–6.4 mm) and large-aperture (6.5–6.8 mm) groups. The differences between the results of the above studies and our study may be related to differences in postoperative review time, preoperative refractive error, and measuring instruments [14]. The increase in HOAs after corneal refractive surgery is significantly correlated with the effective optical zone and corrected refractive error. In this study, the preoperative corrected refractive error did not differ between the 2 groups. Therefore, the difference in the optical-zone diameter may be the main factor affecting the changes in HOAs in the 2 groups.

This study also analyzed the subjective visual quality of patients after SMILE. The results showed that the patients complained of various degrees of discomfort, such as glare, halo, dry eyes, and foreign body sensation, after the surgery. Complaints of obvious binocular foggy vision and poor vision clarity in the early postoperative period may be related to edema of the corneal stroma caused by lens involvement during surgery. Liu et al.[20] showed that both SMILE- and FS-LASIK-affected eyes showed varying degrees of dryness, blurred vision, foreign body sensation, and eye pain in the early postoperative stage, which is similar to our results. In this study, the subjective visual-quality questionnaire scores slightly differed between the two groups at 1 month after the surgery compared with the preoperative scores (*P* > 0.05), and gradually recovered to the preoperative level at 3 months after the surgery. The subjective scores at 1 month after the surgery were slightly higher in the 6.8 mm group than in the 6.5 mm group month (*P* = 0.058, close to 0.05), suggesting that the larger the optical-zone diameter, the better the subjective postoperative visual quality. However, no significant difference in subjective scores was found between the 2 groups at 3 months after the operation. It was considered that the damage caused by the surgery had been repaired, and the adaptability had gradually improved, so the difference in subjective visual quality was not obvious. Symptoms such as glare, halo, and decreased night-vision quality after corneal refractive surgery are related to the effective optical-zone diameter after the surgery, and when the optical-zone diameter is smaller than the diameter of the dark pupil, the symptoms are more obvious [5, 14]. We used a Sirius 3D anterior-segment analysis system to measure the scotopic pupil size in a dark room before the surgery and found that there was no significant difference in scotopic pupil diameter between the two groups. Therefore, the influence of dark pupil diameter on the evaluation of postoperative visual quality was excluded in this study. Zhao et al.[12] also confirmed that the subjective visual-quality scores at 3 months after FS-LASIK did not differ between the 6.0 mm and 6.5 mm optical zone groups, and both groups obtained good postoperative visual quality, which is consistent with our results, and further illustrates that the optical-zone diameter does not greatly impact the subjective visual quality in the long term.

This study further analyzed the correlation of subjective and objective visual quality after SMILE with pupillary diameter and optical-zone diameter, and found that the subjective scores at 1 and 3 months after surgery were significantly and negatively correlated with pupillary diameter (*P* < 0.05). The RMS values of objective visual quality indicators, such as HOAs, spherical aberration, coma, and trefoil, had no significant correlation with pupillary diameter (*P* > 0.05), indicating that the smaller the dark pupil diameter the better the subjective visual quality in the early postoperative period. The relationship between pupillary diameter and night-vision quality is controversial [21–23]. Lin et al. believed that pupillary diameter had no significant relationship with the deterioration of night vision [23]. Chan and Manche [22] found that pupillary diameter had no significant effect on the early postoperative visual symptoms. However, Schallhorn et al.[23]showed that pupillary diameter was related to the occurrence of night visual impairment symptoms such as glare and halo in the early stage after LASIK, but that there was no significant correlation 6 months after the surgery. In this study, the pupillary diameter was significantly and negatively correlated with the subjective visual symptom scores, which supported the effect of increasing pupillary diameter on early postoperative visual quality.

The subjective visual-quality score in the early postoperative period was significantly and positively correlated with the optical-zone diameter (*P* < 0.05), while objective visual-quality parameters such as HOAs, spherical aberration, and coma were significantly and negatively correlated with the optical-zone diameter (*P* < 0.05), indicating that the larger the optical-zone diameter during surgery, the better the subjective and objective visual quality in the early postoperative period. There was no significant correlation between the subjective visual quality scores and optical-zone diameter at 3 months after surgery (*P* > 0.05), which indicated that over time, the adaptability gradually improved, and the effect of optical-zone diameter on the subjective visual quality was not significant.

In conclusion, SMILE in different optical zones has good effectiveness and predictability in the treatment of low and moderate myopia. A larger optical-zone diameter can effectively reduce the degree of postoperative increase in corneal HOAs associated with a pupillary diameter of 6 mm, and yield better visual quality in the early postoperative period. Therefore, for patients with low-to-moderate myopia, the diameter of the target lenticule (optical zone) should be adjusted to improve the subjective and objective visual quality in the early postoperative period. However, in the long term, the 6.5 mm optical zone has no effect on the subjective visual quality of the patients and can save corneal tissue.

### Limitations

(1) This study only observed changes in visual quality within 3 months after the surgery. The follow-up time is short, and the sample size is small. Long-term follow-up after SMILE with different optical-zone diameters in a larger sample of patients is needed. (2) This study did not consider the effect of tear-film stability on visual quality, and research on the effects of dry eye and corneal biomechanical changes is required. (3) The study included only patients with low-to-moderate myopia, and the relationship between optical-zone diameter and postoperative visual quality needs to be studied in patients with high myopia.

## Data Availability

All data generated or analyzed during this study are included in this article. Further enquiries can be directed to the corresponding author.
